# A Case Report about Glycogenic Hepatopathy

**DOI:** 10.1155/2022/5134049

**Published:** 2022-10-18

**Authors:** L. Plaza Enriquez, N. Konindala, H. Yeh, P. Khatiwada, M. Sanchez Valenzuela, K. Askari

**Affiliations:** ^1^Internal Medicine Residency, Memorial Healthcare System, Hollywood, FL, USA; ^2^Internal Medicine Residency, St. Barnabas Hospital, Bronx, NY, USA; ^3^Department of Endocrinology Memorial Healthcare System, Hollywood, FL, USA

## Abstract

**Introduction:**

Glycogenic hepatopathy is a rare complication of uncontrolled diabetes mellitus that presents with hepatomegaly and transient elevation in serum aminotransferase enzymes. The underlying pathophysiology involves excessive accumulation of intrahepatic glycogen. Glycogenic hepatopathy is usually underdiagnosed because it is difficult to differentiate from other entities, such as the nonalcoholic fatty liver. The gold standard for diagnosis is liver biopsy. Glycogenic hepatopathy can be reversed by the achievement of adequate glycemic control. *Case description*. A 19-year-old female patient with a history of poorly controlled type 1 diabetes mellitus that resulted in several episodes of diabetes ketoacidosis requiring hospital admissions. The patient presented to the emergency room with generalized weakness and fatigue found to have diabetic ketoacidosis. Blood tests revealed abnormal liver function with aspartate aminotransferase 1129 U/L (13–37 U/L), alanine aminotransferase 766 U/L (13–56 U/L), alkaline phosphatase 216 U/L (45–117 U/L), total bilirubin 1.0 mg/dL (0.2–1.3 mg/dL), albumin 3.8 g/dL (3.4–5.0 g/dL), partial thromboplastin time < 20 s (23–31 s), prothrombin time 11.8 s (9.5–11.5 s), and international normalized ratio 1.1. Acute hepatitis serologies were negative. Epstein–Barr virus and cytomegalovirus were ruled out. Extensive autoimmune hepatitis tests were negative. Primary biliary cirrhosis was also ruled out. A liver biopsy was obtained, which was diagnostic of glycogenic hepatopathy.

**Conclusion:**

Glycogenic hepatopathy must be suspected in patients with uncontrolled type 1 diabetes mellitus who present with elevated liver enzymes and hepatomegaly. Treating this rare condition requires a timely diagnosis with liver biopsy and strict glycemic control.

## 1. Introduction

Glycogenic hepatopathy (GH) is a rare complication of uncontrolled diabetes mellitus, characterized by hepatomegaly and elevated transaminase levels. It is more commonly reported in the pediatric population with uncontrolled type 1 diabetes mellitus. The pathophysiology of this condition involves excessive accumulation of intrahepatic glycogen, in the presence of high cytoplasmic glucose concentration. GH is usually underdiagnosed because it is difficult to differentiate from other entities, such as nonalcoholic fatty liver (NAFLD), due to overlapping clinical features. The gold standard to make the diagnosis is to perform a liver biopsy with periodic acid-Schiff (PAS) staining, which shows glycogenic deposition within hepatocytes. GH can be reversed by the achievement of tight glycemic control. This condition, however, does not predispose to liver cirrhosis despite the marked elevation of hepatic enzymes and most patients have a good prognosis and complete resolution of symptoms when diagnosed promptly. We present a case of glycogenic hepatopathy in a 19-year-old female patient.

## 2. Case Description

A 19-year-old African American female with a history of type 1 diabetes mellitus presented to the emergency department (ED) with chief complaints of generalized weakness and fatigue for 1 day. She denied abdominal pain, nausea, vomiting, fever, diarrhea, abdominal distention, melena, hematemesis, joint pain, and other symptoms at the time of presentation. Her home medications included 30 units of insulin degludec at bedtime and 5 Units of Insulin Aspart, 15 minutes before a meal. Her total daily dose of insulin was 45 units (total daily dose of insulin 0.85 units/kg/day). The last admission was 4 months ago when she presented with nausea and vomiting and was found to have diabetic ketoacidosis due to medication noncompliance, requiring insulin drip, and ICU admission. She was diagnosed to have type 1 diabetes mellitus at 10 years of age. Her family history was significant for type 2 diabetes in her grandparents. She denied smoking cigarettes, drinking alcohol, and intravenous drug use. Her vital signs upon arrival to ED were temperature-36.6 degree Celsius, pulse-116/min, BP-109/84, respiratory rate-22 breaths/min, and 98% saturation on room air. She was ill-appearing. Physical examination was notable for marked hepatomegaly with liver edge palpable up to 7 cm below the right costal margin. There were no signs of peripheral stigmata of liver disease, and her BMI was 21.48 kg/m^2^.

Initial Laboratory studies were significant for point of care test(POCT) glucose 510 mg/dL (70–90 mg/dL), sodium-130 mmol/L (136–145 mmol/L), potassium 4.9 mmol/L (3.5–5.1 mmol/L) bicarbonate-10 mmol/L (21–32 mmol/L), anion gap-24 mmol/L (5–15 mmol/L), creatinine-0.65 mg/dL, AST-1129 U/L (13–37 U/L), ALT 766 U/L (13–56 U/L), Alkaline Phosphatase 216 U/L (45–117 U/L), Total Bilirubin 1.0 mg/dL(0.2–1.3 mg/dL), albumin 3.8 g/dL(3.4–5.0 g/dL), PTT <20 s (23–31 s), PT 11.8 (9.5–11.5 s), INR 1.1. The beta-hydroxybutyrate level was 8.86 mmol/L (0.02–0.27 mmol/L). ABG showed pH 7.25, pCO2 40 mm Hg (31–45 mm Hg), and pO2 103 mm·Hg (83–108 mm Hg). The urine toxicology screen was negative. The patient received 3 liters of normal saline bolus, regular insulin 6 units of intravenous (IV) bolus in ED. She was admitted to the Intensive Care Unit under diabetic ketoacidosis (DKA) treatment protocol with intravenous insulin drip and IV fluids. She has had multiple prior admissions in our facility related to DKA. This episode was her 5^th^ episode within the past one year. The Hemoglobin A1c on this admission was 12.9%. Her anion gap was closed within 24 hours of presentation with continuous IV insulin drip which was switched to subcutaneous insulin upon anion gap closure.

**Figure 1:**
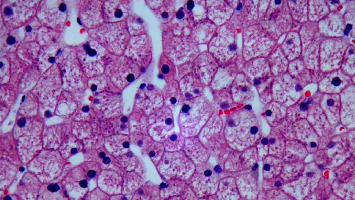
Diffuse hepatocyte enlargement with pale cytoplasm and prominent cytoplasmic membranes. There is a mosaic appearance of the parenchyma due to the compression of sinusoids, resulting in a sheet-like appearance of the hepatocyte architecture. There is no inflammation or fibrosis.

The work-up of the patient's mixed hepatocellular and cholestatic pattern of elevated liver enzymes was done simultaneously along with DKA treatment. She had an elevated gamma-glutamyl transferase(GGT) level of 274 Units/L (5–55 Units/L) confirming the hepatic origin of alkaline phosphatase. Acute hepatitis panel including hepatitis A IgM antibody, hepatitis B surface antigen, core IgM antibody, hepatitis C antibody, EBV, and CMV serologies were negative, mononucleosis screen-negative, and acetaminophen level < 2 ug/mL (10–30 ug/mL). Abdominal ultrasonography showed hepatomegaly measuring 22 cm, diffuse fatty infiltration of the liver, no gallstone, gallbladder distention, gallbladder wall thickening/edema, and normal common bile duct measuring 3 mm in diameter. Ultrasound doppler showed no evidence of Budd–Chiari syndrome or portal vein thrombosis. Workups for autoimmune liver disease including antinuclear antibody (ANA) screen with reflex-specific antiextractable antigen (ENA), anti-smooth muscle antibody, antimitochondrial antibody, liver/kidney microsome (LKM) antibody were all negative. Screening tests for Wilson's disease and hemochromatosis, serum ceruloplasmin, 24-hour urine copper, and serum iron profile, respectively, were obtained, and both were negative. The patient's elevated liver enzymes were thought to be related to hypotension in the setting of DKA and were managed conservatively with intravenous hydration. Despite correction of DKA and hemodynamics, on serial monitoring her liver enzymes continued to remain elevated with AST-1089 U/L (13–37 U/L), ALT 529 U/L (13–56 U/L), alkaline phosphatase 204 U/L (45–117 U/L) on the 4^th^ day of admission. Due to persistently elevated liver enzymes, an ultrasound-guided core needle liver biopsy was performed which showed enlarged and swollen hepatocytes with abundant pale cytoplasm (highlighted with PAS diastase stain), numerous glycogenated nuclei–histologic features consistent with glycogenic hepatopathy. There were no signs of inflammation, the iron stain was negative, no evidence of fibrosis, and fatty change was focal and mild (Figure 1). She tolerated the diet without nausea and vomiting and was discharged home with insulin Lantus 12 units at bedtime and insulin lispro 4 units every meal with close follow-up as an outpatient. However, she had another ED visit presenting with generalized fatigue and was found to have DKA and was admitted to ICU 4 months after this admission.

## 3. Discussion

GH is a rare complication that is predominantly seen in patients with poorly controlled type 1 diabetes mellitus. True incidence and prevalence of GH are unknown, approximately 98% of GH cases have been reported in T1DM [1]. It is characterized by a pathogenic accumulation of glycogen in the hepatocytes causing hepatomegaly and liver enzyme elevation [1]. The pathophysiology involves glucose entry to the hepatocytes through facilitated diffusion and conversion to glucose-6-phosphate by glucokinase. Increased G6P concentration in hepatocytes activates glycogen synthase and further stimulates glycogen synthesis [1].

The presence of insulin promotes glycogenesis by activating glucokinase and glycogen synthase. Hyperglycemia inhibits glycogenolysis by inactivating the phosphorylase which promotes glycogen accumulation in hepatocytes [2]. Patients with diabetic ketoacidosis, hyperglycemia, and high exogenous doses of insulin for the treatment of DKA promote the accumulation of glycogen in hepatocytes. GH is associated with higher A1c levels and recurrent DKA [3].

Common clinical manifestations vary from asymptomatic elevated liver enzymes to abdominal distension, jaundice, and pruritus. A physical exam will reveal hepatomegaly. Although no single serologic test could diagnose GH, elevated transaminases are seen in most patients [1]. Hepatomegaly and elevated transaminases are also common manifestations in patients with NAFLD. It is crucial to distinguish NAFLD from GH since NAFLD is more likely to progress to fibrosis, cirrhosis, and hepatocellular carcinoma [1]. GH is more commonly seen in young poorly controlled type 1 diabetics with multiple DKA episodes, whereas NAFLD is more commonly seen in obese patients with type 2 diabetes mellitus [1]. Glycogen hepatopathy can also present as part of the Mauriac syndrome, a complication of diabetes that consists of delayed puberty, cushingoid features, growth failure, and hepatomegaly. The pathophysiology of the syndrome is thought to be due to impaired glucose utilization in tissues and decreased insulin-like growth factor-1 (IGF-1) and growth hormone levels or receptor resistance to the hormone's action. Mauriac syndrome presents more often in type 1 diabetes but can affect other people, and the sole presentation could be hepatic glycogenosis [4, 5].

CT scans of patients with GH will show increased hepatic attenuation due to increased glycogen deposition, but in patients with NAFLD, the CT will show low attenuation due to increased fat deposition in the liver. MRI with gradient-dual-echo could further differentiate GH and NAFLD by showing low intensity on subtraction in GH and high intensity in NAFLD [6]. However, the definitive diagnosis of GH is made with a biopsy. The classic histologic features include swollen hepatocytes, abundant cytoplasmic glycogen deposits with PAS stain, and minimal portal inflammation, steatosis, or fibrosis [1, 6]. It is imperative to exclude other causes of hepatomegaly and elevated transaminase levels, including autoimmune hepatitis, celiac disease, viral hepatitis, hemochromatosis, and Wilson disease [1].

Our patient had multiple risk factors for GH including long-standing poorly controlled type 1 diabetes, female gender, young age, and recurrent hospital admissions due to DKA since childhood. The rapid influx of glucose into the hepatocytes triggered by treatment with the insulin drip can cause glycogen accumulation in the hepatocytes. Our patient's biopsy revealed classic findings of GH including diffusely swollen hepatocytes with minimal inflammation or fibrosis. The mainstay of management is to improve glycemic control and prevent recurrent episodes of DKA. Resolution of both clinical and biochemical manifestations should be evident after days to weeks with good glycemic control [1].

Some studies reported that continuous insulin infusion may be considered when intensive insulin therapy and a nutritious diet fail to prevent further disease progression [4]. However, in our patient, adequate blood glucose control with a scheduled insulin regimen enabled the reversal of acute elevation of hepatic transaminases.

Approximately 98% of GH cases were reported in type 1 DM and the remaining 2% in type 2 DM. 62% of the reported cases were female, and the remaining 38% were male, indicating a slight female predominance, with most cases occurring in adolescence [1]. Although predominantly found in children and young adults, a case report of glycogenic hepatopathy has been reported in a 29-year-old female by Van den Brand et al. [7], and a 37-year-old male by Sayuk et al. [8]. A review of the literature showed a rare presentation of glycogenic hepatopathy including persistent lactic acidosis and hyperlactatemia [9, 10]. Regan et. Al describes a case of glycogenic hepatopathy with initial improvement in lactic acidosis with the treatment of DKA followed by a sudden unexpected increase. Deemer et. Al shows a case of glycogenic hepatopathy admitted to the Intensive Care Unit with DKA and persistent lactic acidosis and anion gap despite insulin and dextrose infusions. Another interesting case presentation with relapsing hepatitis and associated recurrent pancreatitis, which resolved with tight glycemic control has been reported by Shah et al. [11].

GH is a rare complication mostly seen in poorly controlled type 1 diabetics. Glycogenic hepatopathy should be one of the differential diagnoses in uncontrolled diabetes patients who present with elevated liver enzymes and hepatomegaly. Glycogenic hepatopathy must be differentiated from NAFLD as prognosis differs. Timely diagnosis and treatment could be achieved by aggressively pursuing liver biopsy when suspicion is high and ensuring strict glycemic control after diagnosis. There have been reports of liver fibrosis caused by GH but further studies are required to assess the consequence of the fibrosis identified as severe fibrosis may progress to cirrhosis.

## 4. Conclusion

Glycogenic hepatopathy poses a diagnostic challenge and is a commonly underdiagnosed complication of diabetes mellitus. It is important for clinicians to distinguish this rare disorder from other causes of transaminitis, especially in patients with uncontrolled type 1 diabetes mellitus to ensure timely diagnosis and adequate treatment with improved glycemic control. Further awareness of Glycogenic hepatopathy would help provide earlier diagnosis and treatment to prevent further complications associated with this condition.

## Abbreviations

GH: Glycogenic hepatopathy
NAFLD: Nonalcoholic fatty liver
PAS: Periodic acid-Schiff
AST: Aspartate aminotransferase
ALT: Alanine aminotransferase
ALP: Alkaline phosphatase
PTT: Partial thromboplastin time
PT: Prothrombin time
INR: International normalized ratio
ABG: Arterial blood gas
EBV: Epstein–Barr virus
CMV: Cytomegalovirus.
